# A Resting State Functional Magnetic Resonance Imaging Study of Unmedicated Adolescents With Non-suicidal Self-Injury Behaviors: Evidence From the Amplitude of Low-Frequency Fluctuation and Regional Homogeneity Indicator

**DOI:** 10.3389/fpsyt.2022.925672

**Published:** 2022-06-17

**Authors:** Yi Zhou, Renqiang Yu, Ming Ai, Jun Cao, Xiao Li, Su Hong, Qian Huang, Linqi Dai, LiXia Wang, Lin Zhao, Qi Zhang, Lei Shi, Li Kuang

**Affiliations:** ^1^Department of Psychiatry, The First Affiliated Hospital of Chongqing Medical University, Chongqing, China; ^2^Department of Radiology, The First Affiliated Hospital of Chongqing Medical University, Chongqing, China

**Keywords:** major depressive disorder (MDD), non-suicidal self-injury (NSSI), resting-state functional magnetic resonance imaging (rs-fMRI), adolescents, amplitude of low-frequency fluctuation (ALFF), fractional amplitude of low-frequency fluctuation (fALFF), regional homogeneity (ReHo)

## Abstract

**Background:**

Non-suicidal self-injury (NSSI) behaviors are common in adolescents with major depressive disorder (MDD). Brain studies specifically targeting adolescents with MDD and NSSI may provide new insights into suicide warnings in adolescents with MDD.

**Methods:**

This study examined the whole-brain neural activity in adolescents aged between 12–17 years, 50 unmedicated MDD patients with (nsMDDs) or without NSSI (nnsMDDs), and 25 healthy controls (HCs) participated in this study, and analyzed the correlation between the values of amplitude of low-frequency fluctuation (ALFF), fractional amplitude of low-frequency fluctuation (fALFF) and regional homogeneity (ReHo) in significantly different brain regions and the scores of the relevant clinical psychological scale.

**Results:**

Compared with nnsMDDs, nsMDDs had enhanced ALFF values in left middle occipital gyrus and left median cingulate and paracingulate gyri; the fALFF values of the right caudate nucleus was weakened in the nsMDDs; the ReHo values of right middle occipital gyrus and right middle temporal gyrus weakened and the ReHo values of right medial cingulate gyrus enhanced in nsMDDs. And all of differences were statistically significant. In nsMDDs, the value of ALFF in the left calcarine fissure and surrounding cortex was negatively correlated with the score of Children's depression Inventory (CDI); the value of fALFF in the right caudate nucleus was positively correlated with the score of Beck Scale for Suicidal Ideations (BSSI); the value of ReHo in the right middle temporal gyrus was positively correlated with the score of Multidimensional Anxiety Scale for Children (MASC); and the value of ReHo in the right median cingulate and paracingulate gyri was negatively correlated with the score of BSSI.

**Conclusions:**

We found that in ALFF, fALFF and ReHo, the significant differences between nsMDDs and nnsMDDs are mainly located in default mode network (DMN) and visual network (VN), and there may be brain regions related to NSSI in DMN and VN. The significant differences brain regions in ALFF, fALFF and ReHo between nsMDDs and nnsMDDs were related to the total score of the relevant clinical psychological scale, and may be related to NSSI.

## Introduction

Major depressive disorder (MDD) is a mental disorder characterized by a persistent depressive mood and loss of well-being, loss of interest or pleasure in all activities, and a feeling that life is worthless (1). Between 2005 and 2015, the number of people suffering from MDD increased by 18.4% worldwide. The global rate of MDD is estimated at 322 million people, or 4.4% of the world's population. It has been reported that MDD was the major reason for global non-fatal health loss in 2015, and MDD is predicted to become the greatest burden worldwide by 2030 (2). In recent years, MDD has shown a trend toward a gradual decline with age. The incidence of MDD has gradually increased in the adolescent population, and their prevalence is 20% among all adolescents (3). MDD can have serious consequences, such as self-injury and suicide (4).

Non-suicidal self-injury (NSSI) refers to repetitive behaviors that are not accepted or recognized by the society in which an individual who does not intend to commit suicide causes varying degrees of damage to his or her body (5, 6). A large number of studies have shown that NSSIs are common among adolescents (7–9). Under certain conditions, NSSIs can further aggravate diseases and can even result in suicide (10–12). NSSIs should be recognized a as specific predictor of suicide and a non-specific predictor of psycho-pathological development (13). Self-injury and suicide are major public health problems among adolescents. Self-injury rates are very high among adolescents, and suicide is the second most common cause of death among young people worldwide (14). NSSI is one of the strongest predictors of future suicidal behaviors and has become a public health problem of universal concern (15–17).

In recent years, functional magnetic resonance imaging (fMRI), which has the advantages of non-invasive and non-radiation, plays a more and more important role in the research of mental illness. Many brain area signal abnormalities related to adolescent depression have been found in the study of adolescent depression, but such studies are still lacking, and most of them are based on tasking-state functional magnetic resonance imaging (ts-fMRI). The results may be affected by the differences between the experimental design and the task itself (18). Resting-state functional magnetic resonance imaging (rs-fMRI) provides a non-invasive and non-task method to eliminate some performance-related effects, and provides a reliable method for measuring “baselines” and connections of brain activity (19). Amplitude of low frequency fluctuation (ALFF) is one of the most important indicator in resting state functional magnetic resonance imaging and it is an indicator for measuring resting blood oxygenation level dependent (BOLD) signal changes, which reflects the synchronous cyclic modulation of total cortical excitability and long-distance neurons, and can objectively reflect the brain physiological state of its subjects (20, 21). Although previous studies have certain scientific research value and clinical significance, there is still a lack of research on the abnormality of brain function in adolescent depression from the resting state, in order to more comprehensively understand the brain function activity of adolescent patients with depression. Therefore, this study provides another perspective to explore the abnormal brain activity in adolescent depression with or without NSSI by resting fMRI.

There were few previous studies on MDD adolescents with NSSI, and some brain activity abnormalities related to NSSI were found. Compared with MDD adolescents without NSSI, there were significant abnormalities in amygdala, parahippocampal gyrus, fusiform gyrus, anterior cingulate gyrus and orbital part of frontal gyrus (22, 23). Several other studies found that there were significant differences in amygdala, cingulate gyrus, orbital part of frontal gyrus, precuneus, putamen and striatum in MDD adolescents with NSSI compared those in the with healthy controls (22–26). In a study, compared to healthy control participants, the NSSI group showed decreased amygdala and increased cingulate cortex and orbitofrontal cortex activation to NSSI and negative images (26). In our previous study of depression in MDD adolescents with NSSI, we found that MDD adolescents with NSSI showed significant activation of ALFF signals in the right median cingulate and paracingulate gyri and right fusiform gyrus compared with MDD adolescents without NSSI (27).

The above studies found that the brain regions in default mode network (DMN) are mainly related to NSSI. Most previous studies are based on the exploration of ALFF, with a lack of the analysis of fALFF and ReHo (28). In order to more comprehensively explore the brain abnormalities of adolescent depression patients with NSSI, these two indicators should be added to the data analysis to better explore the differences of local non-parametric consistency in a certain region of the brain and complement each other with ALFF. Thus, this study focuses on the changes of ALFF, fALFF and ReHo in DMN.

The purpose of this study is to explore the brain signal abnormalities of MDD adolescent patients with depression with or without NSSI from the multiple indicators of ALFF, fALFF and ReHo. According to the previous studies and the research results of our research group, the following hypotheses are put forward: (I) Compared with healthy subjects, MDD adolescent patients with or without NSSI show significant differences in ALFF, fALFF and ReHo, and the differences are significant in the brain regions of DMN. (II) Compared with MDD adolescent patients without NSSI, MDD adolescent patients with NSSI show significant differences in ALFF, fALFF and ReHo.

## Methods

A total of 50 right-handed Han adolescents with MDD who were treated at the outpatient clinic of the Department of Psychiatry, the First Affiliated Hospital of Chongqing Medical University, China, were recruited. Their ages were between 12 and 17 years. Twenty five patients (nsMDD group) exhibited NSSI, and 25 patients (nnsMDD group) had no more than 3 NSSI in their lifetime and had no NSSI in the last month. These patients did not take any antidepressants and had not received any psychological or physical therapy before participating in this study.

All of the patients were evaluated by two experienced psychiatrists using the Mini International Neuropsychiatric Interview for Children and Adolescents (MINI-kid) (29), a screening and evaluation tool that conforms to the standards for diagnosing MDD described in the Diagnostic and Statistical Manual of Mental Disorders, the fourth edition (DSM-IV). According to the diagnostic standard for NSSI presented in the Diagnostic and Statistical Manual of Mental Disorders, the fifth edition (DSM-V), only patients who had reported NSSI events lasting 5 days or longer in the past 12 months and at least 1 NSSI in the past month were included in the analysis. The exclusion criteria included a lifetime history of psychiatric illness other than MDD, a history of severe head trauma or neurological illness, a history of any drug or alcohol abuse/dependence at any time, a history of mental illness or suicide in first-degree relatives, other clinically relevant abnormalities in the medical history or laboratory tests and any contraindication for MRI.

In addition, 25 right-handed HC subjects were recruited through advertisements. They were screened with the patient group through the MINI-kid semi-structured clinical interview screening and assessment tool to ensure that subjects with MDD and other mental diseases were excluded from the control group. Exclusion criteria included a history of nervous system illness; severe traumatic brain injury; heart, liver or kidney disease or other serious physical illness; contraindications for any type of MRI scan; substance dependence or abuse; and a history of mental disorders or suicidal behavior among first-degree relatives. All participants underwent additional psychometric testing.

After the completion of this research, all participants received a proper research participation reward.

Before entering the experiment, all the subjects were fully aware of the complete experimental process and their related rights and interests and signed the informed consent form, and all the subjects and their guardians jointly signed the informed consent form. This experiment was approved by the Medical Ethics Committee of The First Affiliated Hospital of Chongqing Medical University (No. 2017–157), and carried out in strict accordance with the experimental procedure.

### Clinical Assessment

The diagnosis of MDD was further confirmed by two senior psychiatrists using the MINI-kid, and the presence of diseases other than MDD, including mania and schizophrenia, was excluded. The Children's depression Inventory (CDI) (30), which has high reliability and validity, was used to evaluate the severity of MDD. Furthermore, all MDD patients underwent an NSSI-related clinical interview. This clinical interview was based on the diagnostic standard recommended by the DSM-V and was used to subjectively and objectively determine whether patients had NSSI, no matter whether they or their parents had reported NSSI or not. To capture the patients' NSSI in as many ways as possible, we asked each recruited patient to complete the Ottawa Self-Injury Inventory (OSI) assessment (31). In addition, the Multidimensional anxiety scale for children (MASC) (32) was used to evaluate the degree of anxiety of patients. The Beck Scale for Suicidal Ideations (BSSI) (33) was used to evaluate patients' self-injury suicidal ideation. The Barratt Impulsiveness Scale (BIS-11) (34), the Connor-Davidson Resilience Scale (CD-RISC) (35) and the Emotion Regulation Questionnaire (ERQ) (36) were used to evaluate the emotion regulation style and behaviors of patients. The Chinese version of the above scale has good reliability and validity in teenagers. And the self-assessments of the adolescents with MDD and the entire clinical assessment process were conducted with the participation of experienced psychiatrists to ensure that the self-assessment data were relatively objective, timely, accurate and complete.

### Acquisition of rs-fMRI Data

All of the recruited adolescents underwent a head MRI examination within 36 hours of the clinical assessment. The majority of the recruited adolescents had never received a head MRI examination; therefore, they were asked to rest for at least 30 minutes between the clinical assessment and the MRI examination. In addition, they were asked to arrive at the waiting area of the examination room 30 minutes in advance to adapt to the environment in order to minimize the first-test effect. During this period, they were asked not to deliberately engage in any activity that involved excessive physical and mental effort. MRI images were acquired through a 3T GE Signa HDxt MRI scanner (General Electric Healthcare, Chicago, Illinois, USA) and an 8-channel head coil. The subjects were asked to remain relaxed, close their eyes, keep their minds clear and avoid thinking as much as possible. None of the participants reported falling asleep during the scan. Appropriate foam pads were used to secure the head to minimize head movement, and comfortable earplugs were used to reduce machine noise and minimize the impact of noise on the examination. The pulse sequence parameters were as follows: repetition time (TR) 2000 ms, echo time (TE) 40 ms, field of view (FOV) 240 × 240 mm^2^, matrix 64 × 64, flip angle 90°, number of slices 33, slice thickness/interval 4/0 mm, scan time 8 min, and volume 240 min. Three-dimensional T1-weighted MRI images were used for rs-fMRI joint registration. The following parameters were recorded: TR 24 ms, TE 9 ms, FOV 240 × 240 mm^2^, matrix 256 × 256, flip angle 90°, and slice thickness/interval 1.0/0 mm.

### Preprocessing of rs-fMRI Data

The quality of the original data is controlled by two experienced imaging professionals to ensure that there is no excessive head movement or abnormal signals in each image, and then classify and sort out the scanned data for pre-processing.

After the classified image data is stored in the form of DPABI software based on MATLAB2018b platform and SPM12 (http://www.fil.ion.ucl.ac.uk/spm) toolkit (DPABI_V5.1 version of https://rfmri.org/dpabi) recognizable data, the data pre-processing goes through the following processes: (1) the original data of structural and functional images are converted into NII and NIFTI formats. (2) the first 10 time points are removed to ensure that all images are not affected by the initial instability of the magnetic field and the first measurement effect of the subjects. (3) the difference method is used to correct the scanning time difference by using the 33rd layer as the reference layer. (4) use Friston-24 model to regress various covariates such as white matter, cerebrospinal fluid and head movement parameters to minimize the impact of physiological noise on data analysis (37). (5) use DARTEL method to register all subjects on the brain template in standard space, and then use its algorithm to transform and resample each subject's brain image to a spatial resolution of 3 × 3 × 3 (mm 3). (6) spatial de-smoothing, the parameters are set to 4 × 4 × 4 (mm3) half width and height to remove the influence of individual extreme signals. (7) use de-linear drift to regress the influence of signals related to the time series of BOLD signals. (8) filter the frequency signals other than 0.01–0.1 Hz to eliminate the signals that may accompany ALFF from the heartbeat, respiratory signals and the noise of the scanner itself, so that the fMRI signals belonging to the subjects' brain can be better analyzed later. (9) check the head motion parameter file. The data of head movement rotation ≤ 3° and translation ≤ 3 mm in three directions (x, y, z) were retained for subsequent data analysis. After the above steps, the data of 75 subjects met the requirements of preprocessing and were included in the follow-up analysis.

### ALFF, fALFF and ReHo Analysis

In the preprocessing step, the eighth filter is removed, and then the operation is carried out based on the ALFF/fALFF module included in the DPABI software. In order to ensure that the signal of the image is based on the signal of the original brain, the results of ALFF and fALFF data are used for analysis. ALFF/fALFF is a commonly used indicator in fMRI, which is based on the fast Fourier transform of each voxel signal of the time series in the whole brain image and converted into the square root of the frequency domain power spectrum. In this study, ALFF represents the average value of each voxel in the frequency range from 0.01–0.1 Hz, while fALFF is based on the ratio of 0.01–0.1 hZ in the full frequency range retained by this study. fALFF is regarded as one of the ways to regress some of the horizontal signals of the whole brain (38). The calculation of ReHo indicator is based on the removal of the sixth de-smoothing in the preprocessing steps, and after the calculation of ReHo indicator, the mean and spatial de-smoothing is carried out to return to a very small number of individual extremes, so as to reduce the false positive signal of a single voxel. ReHo calculation indicator can reflect the synchronization of neurons in the local area, which is widely used in fMRI research, and is more representative (39). Therefore, the calculation of this indicator is included in this study in order to better reflect the brain functional activity of MDD adolescent patients with NSSI.

### Statistical Analysis

In IBM SPSS22.0, Chi-square test was used for the gender data of all subjects included in the study. One-way analysis of variance (ANOVA) was used for age, years of education, age of first episode, total course of disease and clinical scale scores (CDI, MASC, BSSI, CD-RISC, BIS-11 and ERQ). *Post-hoc* Bonferroni tests were performed for each scale score. The significance threshold was set to *p* < 0.05.

In this study, ANOVA was used to evaluate the differences of ALFF, fALFF and ReHo in the brains of the three groups of subjects. If there were statistical differences, two-sample t-test was performed afterwards. The ANOVA was corrected by Gaussian random field theory (gaussian random field, GRF) when calculating the differences between the three groups of ALFF and ReHo. The threshold of voxel was set to 0.05 and the threshold of cluster was set to 0.05 and the one-tail test was used to form the mask brain region after correction. This brain region was used as a restricted statistical analysis area for post-test. When two-sample t-test was performed afterwards, GRF correction was limited to the area of Mask in the previous step (voxel < 0.001, cluster < 0.05, two-tails). Sex, age and mean head movement parameters (mean_FDjenkinson) were taken as covariates when calculating ALFF and ReHo, excluding the possible false results caused by sex, age and head movement.

In addition, we skipped ANOVA and directly used DPABI's own analysis method for *post-hoc* test (Tukey-Kramer) (40). The results showed that DPBAI's own analysis method was more significant in ALFF than in the three groups of brain regions that did not skip ANOVA (cluster contained more voxels), and there was no significant difference between fALFF and ReHo (see the attachment). In this study, we chose not to skip the ANOVA analysis method.

## Results

### Demographic Data and Clinical Characteristics

There was no significant difference between nsMDD, nnsMDD and HC groups in terms of gender (*p* = 0.927), age (*p* = 0.444), education years (*p* = 0.738), ERQ (*p* = 0.129) and cephalomotor parameters [average cephalomotor parameters meanFD-jenkinson (41), *p* = 0.241]. Compared with HC group, nsMDD and nnsMDD groups had higher scores in CDI, MASC and BSSI, and there was no significant difference between the two groups, but in CD-RISC, compared with HC group, nsMDD group and nnsMDD group, the scores were significantly lower, and there was no significant difference between the two groups. It is worth noting that the score of BIS-11 in nsMDD group was significantly higher than that in nnsMDD group and HC group, and there was significant statistical difference, but there was no significant statistical difference between nnsMDD group and HC group. The age of first episode in nsMDD group was earlier than that in nnsMDD group, and the total course of disease in nsMDD group was significantly higher than that in nnsMDD group. The statistical data of the three groups are shown in Table 1.

**Table 1:** Demographic and clinical characteristics and head-motion.

	**nsMDD**	**nnsMDD**	**HC**	**T/F**	**p**	***Post hocs***
	***N* = 25**	***N* = 25**	**N = 25**			
Gender (male/female)	5/20	6/19	6/19	0.152	0.927^a^	-
Age (year)	14.48 ± 1.36	14.96 ± 1.43	14.96 ± 1.77	0.822	0.444^b^	-
Education (year)	8.72 ± 1.65	9.08 ± 1.55	8.96 ± 1.77	0.306	0.738^b^	-
First onset age (year)	12.72 ± 2.48	14.28 ± 1.49	-	0.184	0.010^c^	-
Course (month)	20.08 ± 26.01	8.21 ± 6.19	-	2.176	0.039^c^	-
CDI	34.29 ± 6.71	30.40 ± 4.21	8.10 ± 6.52	76.630	<0.0001^b^	nsMDD>HC, nnsMDD>HC
MASC	76.01 ± 12.31	71.65 ± 11.96	24.10 ± 22.27	50.311	<0.0001^b^	nsMDD>HC, nnsMDD>HC
BSSI	39.64 ± 13.93	30.81 ± 14.47	1.20 ± 2.15	31.676	<0.0001^b^	nsMDD>HC, nnsMDD>HC
CD-RISC	27.67 ± 20.66	32.15 ± 9.62	63.00 ± 10.00	18.908	<0.0001^b^	nsMDD < HC, nnsMDD < HC
BIS-11	100.12 ± 20.08	83.23 ± 15.31	74.70 ± 20.37	8.645	<0.001^b^	nsMDD>nnsMDD, nsMDD>HC
ERQ	39.72 ± 11.20	34.68 ± 11.42	42.60 ± 9.94	2.128	0.129^b^	-
Head motion	0.06 ± 0.03	0.05 ± 0.02	0.06 ± 0.02	1.450	0.241^b^	-

*nsMDD, the depressed adolescent group with non-suicidal self-injury; nnsMDD, the depressed adolescent group without non-suicidal self-injury; HC, the healthy control group. All statistical descriptive parameters are mean ± standard deviation;*

a *passes chi-square test;*

b *one-way ANOVA;*

c *two-samples T-test; pos-hoc, the post-test of one-way ANOVA*.

### ALFF, fALFF and ReHo Results

**Figure 1:**
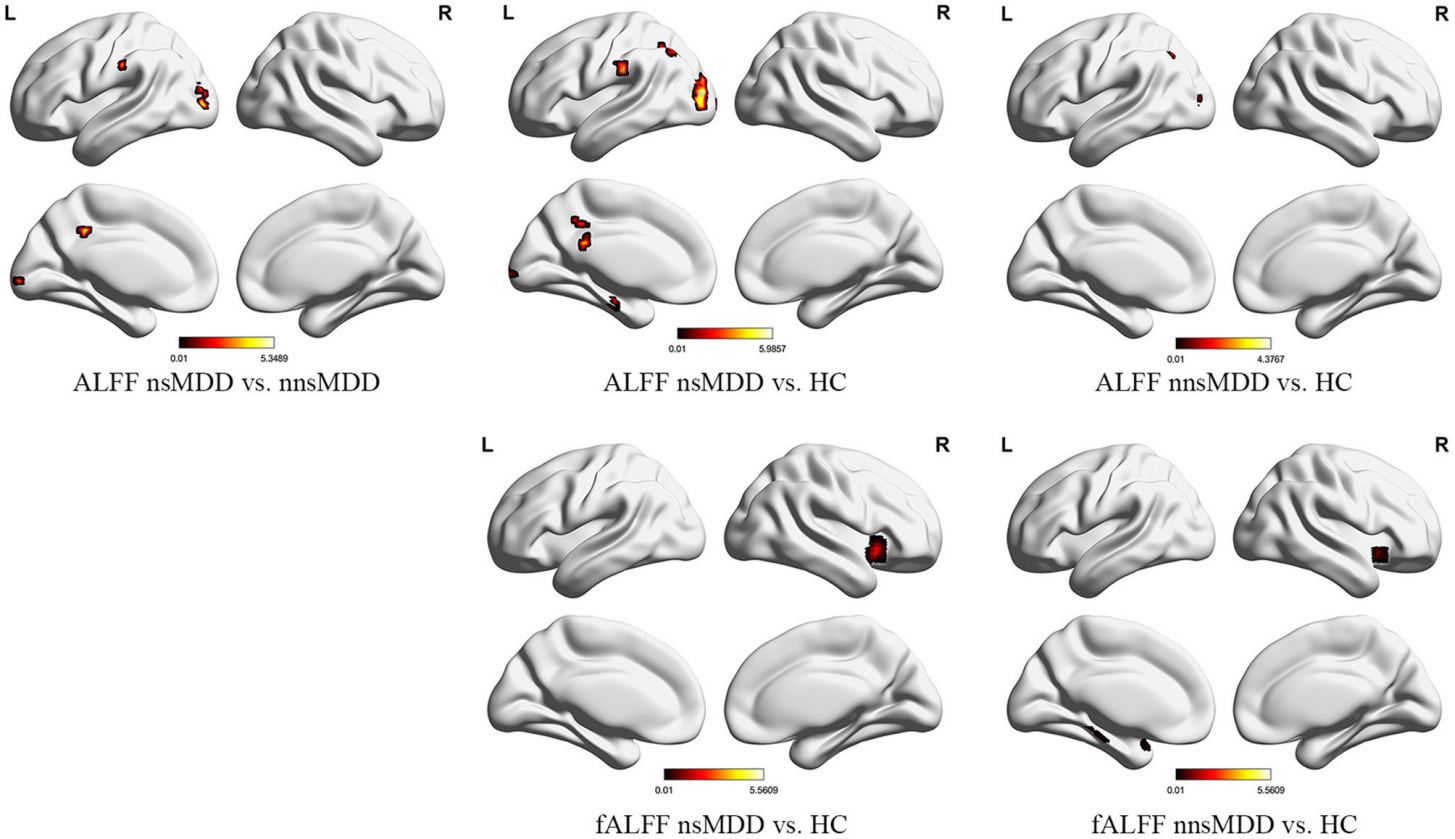
The significant differential brain areas with increased ALFF and fALFF in nsMDD, nnsMDD and HC groups. The figures tool: (https://www.nitrc.org/projects/bnv/).

Compared with nnsMDD group, nsMDD group had enhanced ALFF values in left middle occipital gyrus and left median cingulate and paracingulate gyri. Compared with HC group, nsMDD group had enhanced ALFF values in left middle occipital gyrus, left inferior occipital gyrus, left angular gyrus, left calcarine fissure and surrounding cortex and left parahippocampal gyrus. Compared with HC group, nnsMDD group had enhanced ALFF values in left middle occipital gyrus and left angular gyrus (Figure 1, Table 2).

**Table 2:** Areas with increased or decreased ALFF in groups.

**Region (peak)**	**AAL**	**X**	**Y**	**Z**	**t**	**Voxel**
**nsMDD vs. nnsMDD**						
Occipital_Mid_L	51	−33	−87	3	5.15971	19
Cingulum_Mid_L	33/67/35	−9	−42	39	5.34890	18
Occipital_Mid_L	51	−24	−87	15	3.81805	4
Calcarine_L	43	−9	−96	0	3.69098	2
SupraMarginal_L	63	−60	−24	33	3.76369	2
Occipital_Mid_L	51/61	−36	−69	39	4.20263	2
**nsMDD vs. HC**						
Occipital_Mid_L	51/49	−33	−90	12	5.98572	80
SupraMarginal_L	53/57	−60	−27	27	4.77878	29
Angular_L	65/61/51	−42	−63	42	5.79640	22
Calcarine_L	43/51/47/49	−15	−93	0	4.46443	18
Hippocampus_L	39/37/55	−24	−12	−21	4.08006	11
Cingulum_Post_L	35	−3	−39	24	4.53527	7
Precuneus_L	67/33	−6	−42	39	4.72634	7
Precuneus_L	67	0	−57	51	4.41656	7
**nnsMDD vs. HC**						
Occipital_Mid_L	51	−36	−93	6	4.37670	6
Angular_L	65	−42	−63	42	4.01027	3

*AAL, Automated anatomical labeling; X Y Z, montreal neurological institute (MNI); Sup, superior; Inf, Inferior; Mid, middle; Post, posterior; L, left; R, right*.

**Figure 2:**
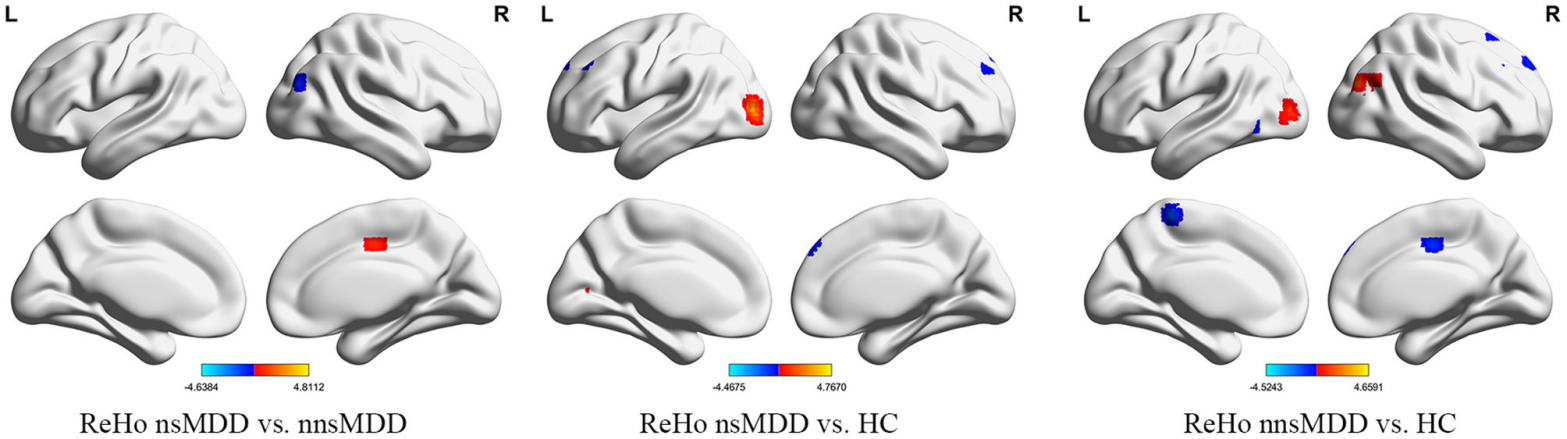
The significant differential brain areas with increased or decreased ReHo in nsMDD, nnsMDD and HC groups. The figures tool: https://www.nitrc.org/projects/bnv/.

Compared with the nnsMDD group, the fALFF values of the right caudate nucleus was weakened in the nsMDD group. Compared with the HC group, the nsMDD group had enhanced the fALFF values of the right insula. Compared with the HC group, the nnsMDD group had enhanced the fALFF values of the right insula (Figure 2, Table 3).

**Table 3:** Areas with increased or decreased fALFF in groups.

**Region (peak)**	**AAL**	**X**	**Y**	**Z**	**t**	**Voxel**
**nsMDD vs. nnsMDD**						
Caudate_R	72	12	−3	18	−3.63883	3
**nsMDD vs. HC**						
Insula_R	30	36	15	−9	5.56086	18
Temporal_Sup_R	82	45	0	−9	3.65109	1
Insula_R	30	45	−3	3	3.56323	1
Hippocampus_L	37	−24	−36	6	3.95574	1
**nnsMDD vs. HC**						
Insula_R	30	36	15	−9	4.00475	8
Thalamus_R	78	9	−15	12	3.75248	3
ParaHippocampal_L	39	−15	0	−24	3.96507	2
Temporal_Pole_Sup_L	83	−36	9	−24	3.90666	2
ParaHippocampal_L	39	−21	−27	−18	3.91029	2
ParaHippocampal_L	39	−15	−6	−24	3.69830	1
Fusiform_L	55	−18	−36	−15	3.67418	1
Temporal_Sup_R	82	48	−12	3	3.63470	1
Caudate_R	72	15	−12	21	3.60546	1

*AAL, Automated anatomical labeling; X Y Z, montreal neurological institute (MNI); Sup, superior; Inf, Inferior; Mid, middle; Post, posterior; L, left; R, right*.

**Figure 3:**
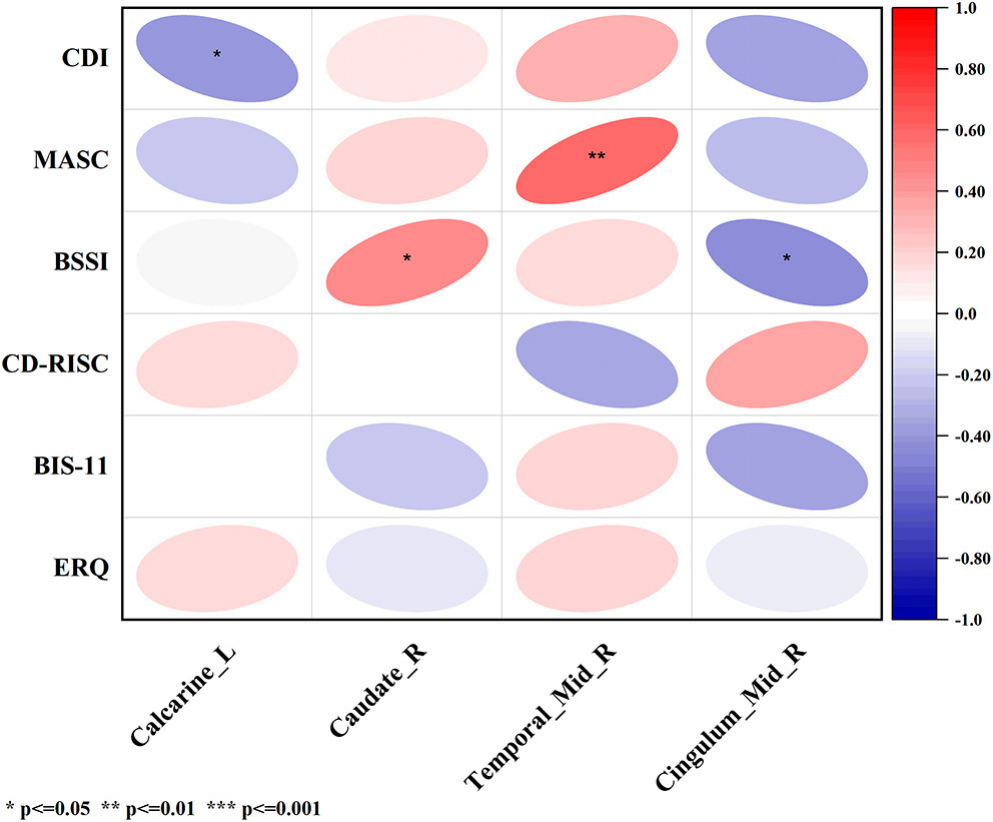
The correlation between ALFF, fALFF and ReHo in the altered brain regions in nsMDD group compared with nnsMDD group and clinical characteristics. The figure tool: www.OriginLab.com. OriginPro Learning Edition (2019110152@stu.cqmu.edu.cn).

Compared with nnsMDD group, the ReHo values of right middle occipital gyrus and right middle temporal gyrus weakened and the ReHo values of right medial cingulate gyrus enhanced in nsMDD group. Compared with the HC group, the ReHo values in the left middle occipital gyrus was enhanced and the ReHo values in the right medial superior frontal gyrus and left middle frontal gyrus weakened in the nsMDD group. Compared with the HC group, the ReHo values of the right middle occipital gyrus was enhanced and the ReHo values of the left precuneus was weakened in the nnsMDD group. And all of differences were statistically significant (Figure 3, Table 4).

**Table 4:** Areas with increased or decreased ReHo in groups.

**Region (peak)**	**AAL**	**X**	**Y**	**Z**	**t**	**Voxel**
**nsMDD vs. nnsMDD**						
Temporal_Mid_R	86	60	−66	21	−3.68597	7
Occipital_Mid_R	52	42	−81	27	−3.62894	6
Cingulum_Mid_R	34	12	−12	42	4.81121	5
**nsMDD vs. HC**						
Occipital_Mid_L	51	−33	−87	9	4.76701	32
Frontal_Sup_Medial_R	24/23	6	48	45	−3.53581	23
Frontal_Mid_L	7	−33	45	39	−3.52675	12
Calcarine_L	43	−18	−75	12	4.01667	3
Frontal_Sup_R	4	24	45	42	−3.53249	3
Frontal_Sup_L	3	−15	54	39	3.53905	1
Frontal_Sup_R	4	24	39	54	−3.53939	1
**nnsMDD vs. HC**						
Occipital_Mid_R	52/86	48	−78	24	4.65908	30
Paracentral_Lobule_L	67/69	−9	−36	66	−3.55170	12
Occipital_Mid_L	51	−36	−90	6	4.03762	9
Cingulum_Mid_R	34	12	−9	42	−3.59837	6
Frontal_Sup_R	24/4	15	48	39	−3.58942	4
Frontal_Mid_R	8	45	33	42	−3.72898	4
Occipital_Mid_L	51	−45	−87	0	4.16002	2
Temporal_Mid_R	86	60	−66	21	3.73527	2
Frontal_Sup_Medial_L	23	0	54	42	−3.59599	2
Frontal_Sup_R	4	24	21	63	−3.52180	2
Temporal_Inf_L	89	−45	−60	−6	−3.61300	1

*AAL, Automated anatomical labeling; X Y Z, montreal neurological institute (MNI); Sup, superior; Inf, Inferior; Mid, middle; Post, posterior; L, left; R, right*.

### Correlation of ALFF, fALFF and ReHo With Clinical Scale Scores

In nsMDD group, the value of ALFF in brain region 4 (the left calcarine fissure and surrounding cortex), which was significantly different from that of nnsMDD group, was negatively correlated with the total score of Children's depression Inventory (CDI); the value of fALFF in brain region 1 (the right caudate nucleus), which was significantly different from that of nnsMDD group, was positively correlated with the total score of Beck Scale for Suicidal Ideations (BSSI); the value of ReHo in brain region 1 (the right middle temporal gyrus), which was significantly different from that of nnsMDD group, was positively correlated with the total score of Multidimensional Anxiety Scale for Children (MASC); and the value of ReHo in brain region 3 (the right median cingulate and paracingulate gyri), which was significantly different from that of nnsMDD group, was negatively correlated with the total score of BSSI (Figure 3, Tables 5, 6).

**Table 5:** The brain regions of interest for correlation analysis with clinical characteristics.

**Indicator**	**ROI**	**Region (peak)**	**AAL**	**X**	**Y**	**Z**	**t**	**Voxel**
**nsMDD vs. nnsMDD**								
ALFF	N0.1	Occipital_Mid_L	51	−33	−87	3	5.15971	19
ALFF	N0.2	Cingulum_Mid_L	33/67/35	−9	−42	39	5.34890	18
ALFF	N0.3	Occipital_Mid_L	51	−24	−87	15	3.81805	4
ALFF	N0.4	Calcarine_L	43	−9	−96	0	3.69098	2
ALFF	N0.5	SupraMarginal_L	63	−60	−24	33	3.76369	2
ALFF	N0.6	Occipital_Mid_L	51/61	−36	−69	39	4.20263	2
fALFF	N0.1	Caudate_R	72	12	−3	18	−3.63883	3
ReHo	N0.1	Temporal_Mid_R	86	60	−66	21	−3.68597	7
ReHo	N0.2	Occipital_Mid_R	52	42	−81	27	−3.62894	6
ReHo	N0.3	Cingulum_Mid_R	34	12	−12	42	4.81121	5

*ROI, region of interest; AAL, Automated anatomical labeling; X Y Z, montreal neurological institute (MNI); Sup, superior; Inf, Inferior; Mid, middle; Post, posterior; L, left; R, right*.

**Table 6:** Pearson's correlations between ALFF, fALFF and ReHo in the altered brain regions in nsMDD group compared with nnsMDD group and clinical characteristics.

**Indicators**	**ROI**		**CDI**	**MASC**	**BSSI**	**CD–RISC**	**BIS−11**	**ERQ**
ALFF	Calcarine_L (NO.4)	r	−0.412*	−0.223	−0.042	0.164	0.021	0.176
		p	0.045	0.295	0.845	0.444	0.922	0.409
fALFF	Caudate_R (No. 1)	r	0.133	0.186	0.463*	0.033	−0.223	−0.112
		p	0.536	0.383	0.023	0.877	0.296	0.601
ReHo	Temporal_Mid_R(No. 1)	r	0.330	0.600**	0.162	−0.356	0.191	0.188
		p	0.115	0.002	0.449	0.088	0.370	0.380
ReHo	Cingulum_Mid_R(No. 3)	r	−0.372	−0.280	−0.456*	0.365	−0.380	−0.085
		p	0.073	0.185	0.025	0.079	0.067	0.693

* *p < = 0.05*

** p < = 0.01 ***p < =0.001; ROI, region of interest; Sup, superior; Inf, Inferior; Mid, middle; Post, posterior; L, left; R, right.

## Discussion

In this study, it was found that the differences between nsMDD and nnsMDD in ALFF, fALFF and ReHo were located in DMN and visual network (VN) as well as a small number of salience network (SN) and sensorimotor network (SMN). There may be brain regions closely related to NSSI in DMN, VN, SN and SMN brain networks. Compared with the HC group, there were significant differences in the brain regions of nsMDD in DMN, VN and a small amount of central executive network, which confirms with previous studies (22–27).

There was a significant difference in the total score of the BIS-11 between the nsMDD group and the nnsMDD group. The score of the nsMDD group was significantly higher than that of the nnsMDD group. The higher the score of the BIS-11 was, the more impulsive and impulsive the subjects would be to deal with things. The score of the BIS-11 of MDD adolescents with NSSI was significantly higher than that of MDD adolescents without NSSI, which is consistent with previous studies. The related BOLD signals on fMRI were significantly different. The ALFF was significantly enhanced in the left middle occipital gyrus and medial lateral cingulate gyrus, while the ReHo was significantly weakened in the right middle temporal gyrus, middle occipital gyrus and medial lateral cingulate gyrus. This indicates that the signal abnormalities in these brain regions may be closely related to NSSI. Some scholars have pointed out that the level of impulsive behavior can be used as a predictor of NSSI in MDD adolescents with NSSI (42). In the future, the score of BIS-11 and bilateral middle occipital gyrus and medial lateral cingulate gyrus may be used as focus on brain areas to further explore the situation of MDD adolescents with NSSI before and after treatment, in order to further discover the pathophysiological mechanism closely related to NSSI. In this study, obvious abnormalities were only found in ALFF and ReHo between the two patient groups, and the differences in ALFF and ReHo in this study were also found to be fewer voxels. Although corrected by multiple comparisons, a larger sample size is still needed to further verify in the future, and then further explore the abnormal areas of the brain that may be related to NSSI.

Due to the limited number of cases collected and because the age of the first episode and the total course of disease could not be easily controlled, subjects were thus not more carefully grouped to explore the abnormal BOLD signal of the brain of MDD adolescents with or without NSSI. Although no obvious abnormality of brain BOLD signal was found in MDD adolescents with or without NSSI in this study, there were still significant differences in ALFF, fALFF and ReHo between these two patient groups and HC group, which is consistent with the previous research in our group (43–45), which further confirms that the brain network closely related to MDD may be related to DMN and SN. Some related studies have shown that there is a close relationship between DMN and SNs and cognition, while the NSSI of MDD adolescents with NSSI may be closely related to the level of self-awareness. We can thus infer that NSSI may be closely related to DMN and SN. In the future, we can combine a variety of data analysis methods (such as graph theory combined with data-driven independent component analysis) to further explore the abnormal activity of DMN and SN in the brain functional network of MDD adolescents in NSSI.

The difference between this study and other studies lies in that, in the correlation analysis with the multiple clinical psychological scales, each cluster was analyzed separately with the total score of the scale in order to further clarify the specific clusters related to the differential brain regions and the total score of the scale. In this study, it was found that ALFF, fALFF and ReHo signals in different brain regions of nsMDD and nnsMDD groups were correlated with the total scores of CDI, MASC and BSSI. There was a negative correlation between the fourth area of interest in ALFF (mainly in the left calcarine fissure and surrounding cortex, belonging to the VN) and the total score of CDI (Figure 3, Tables 5, 6). Although there were only two voxels, it could still reflect to some extent that the brain of MDD adolescents may be closely associated with depression-related symptoms in the VN. The lower the ALFF signal in the left calcarine fissure and surrounding cortex may indicate more severe depressive symptoms in MDD adolescents. So far, few studies have found that there is a correlation between VN brain area and MDD and NSSI in adolescents. In the future, we can further explore whether there is a significant correlation between VN brain area and NSSI. In the different brain regions of the fALFF indicators of the two patients, it was found that there was a positive correlation between the brain region of interest No 1 (mainly in the right caudate nucleus, belonging to the SN) and the total score of BSSI (Figure 3, Tables 5, 6). There were only 3 voxels, but it can be inferred from this correlation analysis that the fALFF signal value in the brain region of the SN, especially in the right caudate nucleus, was positively correlated with suicidal ideation (post-test). There was no significant difference in the total score of BSSI between nsMDD group and nnsMDD group, but the average score of nsMDD group was higher than that of nnsMDD group. We can speculate that in MDD adolescents, the higher the fALFF signal in the right caudate nucleus is, the more likely it is to have NSSI. In addition, in the ReHo indicator, there was a significant correlation between the differential brain regions between nsMDD and nnsMDD groups and the total scores of MASC and BSSI scales. The signal value of the first region of interest (right middle temporal gyrus, belonging to SMN) in the differential brain regions of ReHo was positively correlated with the total score of MASC (Figure 3, Tables 5, 6), and in the third brain area of ReHo differential brain regions (right medial cingulate gyrus). There was a negative correlation between the signal value of SMN and the total score of BSSI (Figure 3, Tables 5, 6). It can be inferred that the higher the ReHo signal value of the right middle temporal gyrus in the SMN of the brain is, the severer their anxiety symptoms are, and the lower the ReHo signal value of the right medial cingulate gyrus in the SMN of the brain indicates that their suicidal ideation is more obvious and NSSI is more likely to occur. It is worth noting that there was no significant correlation between the differential brain regions of ALFF, fALFF and ReHo and the total score of clinical psychological scale between the two case groups and the healthy control group, respectively. It is possible that in the resting state functional magnetic resonance imaging, MDD adolescents with or without NSSI have no specific brain regions related to the total score of each clinical psychological scale in this study, and may not be found to be true positive because of the small sample size (46).

## Conclusion

In this study, it was found that in the indicators of ALFF, fALFF and ReHo, MDD adolescents with or without NSSI may be closely related to DMN and SN compared with adolescents with physical and mental health. In the correlation analysis with the clinical psychological scale, the differences of the local indicators of ALFF, fALFF and ReHo in the resting state fMRI of the nsMDD, nnsMDD and HC groups were compared with the clinical psychological scale. The correlation analysis between the brain regions and the clinical psychological scale showed that the ALFF signal value in the brain area of the VN (especially in the left calcarine fissure and surrounding cortex) of MDD adolescents was significantly lower than that of normal adolescents, which may indicate that they have more serious depressive symptoms. NSSI is more likely to occur. The significant increase of fALFF signal value in the brain region of SN (especially the right caudate nucleus) may indicate that it is more likely to have NSSI and have stronger suicidal ideation, while the significant abnormality of ReHo signal value in the brain region of SMN (especially in the right middle temporal gyrus and right medial cingulate gyrus) indicates that NSSI, severer anxiety symptoms and stronger suicidal ideation are more likely to exist.

## Data Availability Statement

The original contributions presented in the study are included in the article/Supplementary Material, further inquiries can be directed to the corresponding author/s.

## Ethics Statement

The studies involving human participants were reviewed and approved by Ethics Committee of the First Affiliations Hospital of Chongqing Medical University (No. 2017-157). Written informed consent to participate in this study was provided by the participants' legal guardian/next of kin. Written informed consent was obtained from the minors' legal guardian/next of kin for the publication of any potentially identifiable images or data included in this article.

## Author Contributions

YZ designed the research, collected samples, analyzed data, and wrote the original of manuscript. RY collected samples, supervised, and conducted quality control. MA, JC, XL, and SH supervised data and conducted quality control. QH, LD, and LW collected samples and gave some advice. LZ supervised the research, gave some advice, and revised the manuscript. QZ and LS collected samples and gave some advice. LK supervised the research, provided funding, and gave some advice. All authors contributed to the article and approved the submitted version.

## Funding

This work was supported by the National Natural Science Foundation of China (Grant nos. 81671360 and 81971286).

## Conflict of Interest

The authors declare that the research was conducted in the absence of any commercial or financial relationships that could be construed as a potential conflict of interest.

## Publisher's Note

All claims expressed in this article are solely those of the authors and do not necessarily represent those of their affiliated organizations, or those of the publisher, the editors and the reviewers. Any product that may be evaluated in this article, or claim that may be made by its manufacturer, is not guaranteed or endorsed by the publisher.
